# Two hits are better than one: synergistic anticancer activity of α-helical peptides and doxorubicin/epirubicin

**DOI:** 10.18632/oncotarget.2754

**Published:** 2014-12-19

**Authors:** Jing Zhao, Yibing Huang, Dong Liu, Yuxin Chen

**Affiliations:** ^1^ Key Laboratory for Molecular Enzymology and Engineering of the Ministry of Education, Jilin University, Changchun, China; ^2^ National Engineering Laboratory for AIDS Vaccine, Jilin University, Changchun, China; ^3^ School of Life Sciences, Jilin University, Changchun, China

**Keywords:** Anticancer peptides, doxorubicin/epirubicin, synergy, mechanism of action, xenograft model

## Abstract

This study explored combinational anticancer therapy using α-helical peptides HPRP-A1/HPRP-A2 with the chemical drugs doxorubicin (DOX) and epirubicin (EPI). The *in vitro* activity of these drugs against different cancer cell lines was synergistically increased, as was their activity in a HeLa xenograft model in BALB/c nude mice. We delineated the mechanism of this synergy by studying the apoptosis pathway and morphologic changes in the HeLa cell membrane. The mechanism of the HPRP-A1/DOX combination was found to involve enhanced apoptosis, which seemed to be caspase-dependent and involved both the extrinsic and intrinsic parts of the caspase cascade in HeLa cells. Combined application of HPRP-A1 and DOX at low concentrations was significantly more effective than either drug alone against HeLa tumors in the mouse xenograft model. This type of combination therapy appears to have great clinical potential.

## INTRODUCTION

Although breakthroughs in tumor treatment are frequently reported, severe toxicity in normal cells and low efficacy against multidrug-resistant cancer cells still preclude the successful development of new conventional anticancer drugs for clinical use [1, 2]. However, to overcome adverse drug reaction, novel approaches for cancer therapy are urgently required. Doxorubicin (DOX) and epirubicin (EPI) are stereoisomers with a broad spectrum of antitumor activity and are taken up by tumor cells through slow passive diffusion across the plasma membrane. However, DOX has a disappointing 30% overall response rate, which is a major factor in limiting its uptake into tumor cells [3, 4]. There has been much research interest into the design of nanocarriers and hydrophilic peptide conjugants for anticancer drugs [5, 6]. However, these carriers often have limited drug loading capacity and are thus unlikely provide a significant clinical improvement [7]. Sugahara et al. showed that co-administration of iRGD (a tumor-penetrating peptide) with different types of cancer drugs was slightly more effective than the conjugated drug at inhibiting tumor growth and tumor accumulation [8]. In combination therapy, the effective cytotoxic doses of chemotherapeutic drugs are dramatically reduced with a concomitant decrease in adverse events, so this strategy represents a superior approach to the use of single drugs [4, 9, 10]. Multi-component therapeutics with several compounds that interact with diverse targets has become a renewed research focus [11]. Currently, nearly all successful cancer chemotherapy regimens are combinations of multiple agents given simultaneously, thereby achieving better therapeutic efficacy and minimizing side effects [9].

Many studies have shown that some synthetic and natural cationic peptides exhibit anticancer activity, including rapid cytotoxicity, a broad spectrum of activity and high specificity for cancer cells [12, 13]. The clinical anticancer potential of cationic peptides derives from their targeting of the cytoplasmic membrane, allowing them to bypass the cellular mechanisms of multidrug resistance and produce membrane lysis or increased permeabilization [14, 15]. Some cationic peptides (such as defensins and cecropins) not only induce cell death (by increasing membrane permeability leading to cell lysis and/or changes in membrane barrier function) but also have the potential to enhance the efficacy of different chemotherapeutics against multidrug-resistant tumor cells [16]. Furthermore, some cationic peptides are highly specific towards tumor cells rather than normal cells [17–19]. Hence, these cationic peptides may be an important complementary adjunct to conventional chemotherapeutics.

In this study, we hypothesized that cell membrane disruption by α-helical anticancer peptides should increase the intracellular concentration of a conventional chemotherapeutic agent given simultaneously, thus enhancing its anticancer effect. We systematically studied the synergistic effect between α-helical peptides and conventional chemotherapeutic drugs. HPRP-A1 and its enantiomer HPRP-A2 consist of 15 all-L- or all-D-amino acids, respectively. These peptides were employed together with DOX and EPI to investigate their combined efficacy *in vitro* and *in vivo* and to delineate the mechanism of their synergistic action. The objectives of this study were three-fold: first, to explore the combined anticancer activity of peptides and chemotherapeutic drugs; second, to understand the synergistic mechanism of these two types of anticancer agents; and third, to verify the clinical potential of this new high efficacy, low toxicity approach to cancer chemotherapy.

## RESULTS

### Peptides and anticancer activity

**Figure 1:**
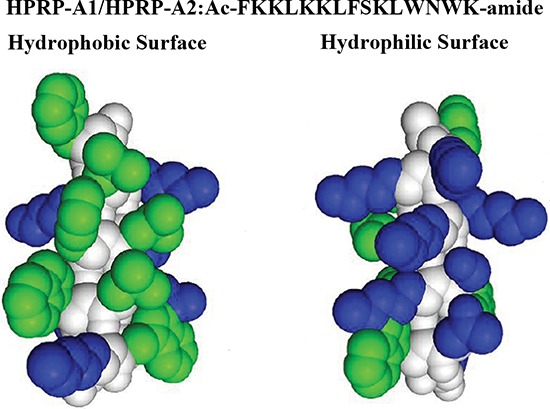
Peptide sequence and space-filling model of HPRP-A1/HPRP-A2

As shown in Fig. 1, peptide HPRP-A1 is a 15-residue α-helical amphipathic membrane-active peptide. Compared with the N-terminus of ribosomal protein L1 (RpL1) of *Helicobacter pylori*, HPRP-A1 shows more than 86% homology in amino acid sequence and forms an amphipathic α-helical structure in the hydrophobic environment with complete polar and non-polar faces [20]. Composed of all D-amino acids, HPRP-A2 is the enantiomer of HPRP-A1. HPRP-A1/HPRP-A2 showed strong anticancer activity and low toxicity against human red blood cells (Table 1). Although the anticancer efficacy of the peptides was inferior to that of the chemical drugs, their greater molecular weight facilitated much faster cytotoxicity, indicating that their mechanism of action was different to the chemical drugs. Similar IC_50_ values between the two enantiomeric peptides indicated that there was no stereochemical effect in the anticancer action of HPRP-A1/HPRP-A2. The all-D peptide has comparable anticancer activity to the all-L analog, indicating that these two α-helical peptides do not act through interaction with a chiral center, such as a receptor or enzyme.

**Table 1:** Anticancer (IC_50_) and hemolytic (MHC) activities of drugs against cancer cells and human red blood cells

Drugs	IC_50_^a^(μg/ml)			MHC^b^ (μg/ml)
	24 h		1.5 h	
	HepG2	HeLa	HeLa	
**HPRP-A1**	27.05 ± 0.13	25.22 ± 0.18	25.52 ± 0.11	154.7 ± 4.32
**HPRP-A2**	23.56 ± 0.11	25.85 ± 0.23	23.40 ± 0.09	148.3 ± 5.37
**EPI**	1.98 ± 0.22	1.55 ± 0.17	> 500	—
**DOX**	2.38 ± 0.15	2.14 ± 0.24	> 500	—

__: Data not tested.

a Anticancer activity (IC_50_) represents the concentration of drug at which cell viability was reduced by 50% compared with untreated cells.

b Hemolytic activity (MHC) was determined on human red blood cells after incubating with peptides for 1.5 h.

### Co-treatment induces HeLa/HepG2 cell death *in vitro*

**Figure 2:**
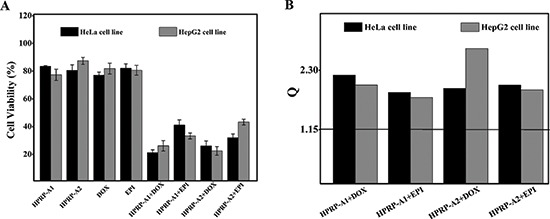
Cell viability and combination index of HeLa and HepG2 treated with drug combination **Panel A**: Growth inhibition in HeLa and HepG2 cells after incubation for 24 h with a combination of HPRP-A1/HPRP-A2 (16 μg/ml) and DOX/EPI (1.2 μg/ml). Results are expressed as percentage of the control ± SD of three independent experiments. **Panel B**: combination index (Q) of the combination treatment of HPRP-A1/HPRP-A2 and DOX/EPI, where *Q* < 0.85, *Q* > 1.15 and 0.85 < *Q* < 1.15 indicate antagonism, synergy, and additive effect, respectively.

Two well-studied cell lines (HeLa and HepG2) were selected to study whether low-dose drug combinations produces synergistic effects on cell growth *in vitro*. Cells were treated with HPRP-A1/HPRP-A2 alone (16, 12 and 8 μg/ml), DOX/EPI alone (1.2, 0.8 and 0.4 μg/ml) and various peptide-drug combinations (HPRP-A1/DOX, HPRP-A1/EPI, HPRP-A2/DOX and HPRP-A2/EPI) for 24 h. MTT assays were used to evaluate the effects of these combinations on cell growth. The drug concentrations used were selected based on the IC_50_ values of each drug alone (Table 1). At sub-threshold doses, there was no distinct cytotoxicity or growth reduction when used alone. However, these same drug doses used in the peptide-drug combinations (HPRP-A1/DOX, HPRP-A1/EPI, HPRP-A2/DOX, and HPRP-A2/EPI) produced significant cytotoxicity (Fig. 2A and Supplementary Fig. S1). According to the Jin's formula [21], all Q (combination index) values were >1.15, which indicates that there were significant synergistic effects between the α-helical peptides and the conventional anticancer drugs in both cell lines (Fig. 2B). Because the chemical drugs had similar anticancer activity in combination with both peptide stereoisomers (HPRP-A1/HPRP-A2 or DOX/EPI), we used only HPRP-A1 and DOX in subsequent experiments.

### Combination with HPRP-A1 enhanced cellular uptake of DOX

**Figure 3:**
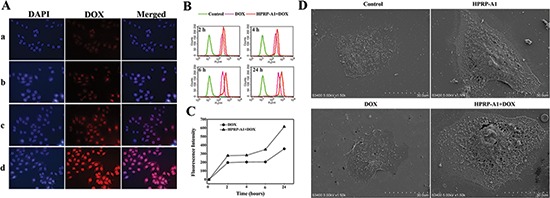
Fluorescence and flow cytometry studies of cellular entry of drugs of HeLa cells treated with DOX alone or DOX and HPRP-A1 combination, and morphological study of HeLa cells treated with different drugs by scanning electron microscopy **(A)** Representative images (400× magnification) of HeLa cells incubated with DOX or DOX+HPRP-A1 at different time intervals: a. DOX (15 μg/ml), 1 h; b. DOX (15 μg/ml) and HPRP-A1 (12 μg/ml), 1 h; c. DOX (15 μg/ml), 3 h; d. DOX (15 μg/ml) and HPRP-A1 (12 μg/ml), 3 h. In panel A, the images from left to right show differential contrast image: cell nuclei stained by DAPI (blue), DOX fluorescence (red), and merged images. **(B)** Time course of DOX accumulation in HeLa cells exposed to DOX (1.0 μg/ml) or DOX (1.0 μg/ml)+HPRP-A1 (12 μg/ml) for 2, 4, 6 and 24 h, measured using flow cytometry. **(C)** Quantitative comparison of fluorescence intensity at 2, 4, 6 and 24 h. **(D)** Scanning electromicroscopic images of HeLa cells treated with HPRP-A1 and/or DOX.

Time-dependent intracellular accumulation of DOX fluorescence was detected by fluorescence microscopy and by flow cytometry using HeLa cells. As clearly shown in Fig. 3A, intracellular DOX fluorescence (red) was more intense in cells incubated with a combination of HPRP-A1 and DOX than with DOX alone after incubation for 1 h and 3 h, respectively. These findings were further confirmed by flow cytometry (Fig. 3B). As expected, the flow cytometric histogram of the cells incubated with HPRP-A1+DOX revealed higher fluorescence intensity than with DOX alone after incubation at all measured time points (2, 4, 6 and 24 h). The corresponding flow cytometric quantitative comparison of fluorescence intensity in Geomean at these different incubations showed a similar trend, namely that combining HPRP-A1 and DOX enhances cellular uptake of DOX compared with DOX alone (Fig. 3C).

The mechanism of drug uptake into cells was further analyzed by scanning electron microscopy. As showed in Fig. 3D, untreated HeLa cells exhibited an adherent smooth surface. In contrast, pores are visible on the surface of cells after treatment with a combination of HPRP-A1/DOX. Furthermore, while treatment with HPRP-A1 or DOX alone caused a small effect on the integrity of the cell membrane, combination therapy resulted in a severely disrupted cell membrane with significant cavity formation and loss of microvilli and membrane integrity.

### Apoptosis of cancer cells

**Figure 4:**
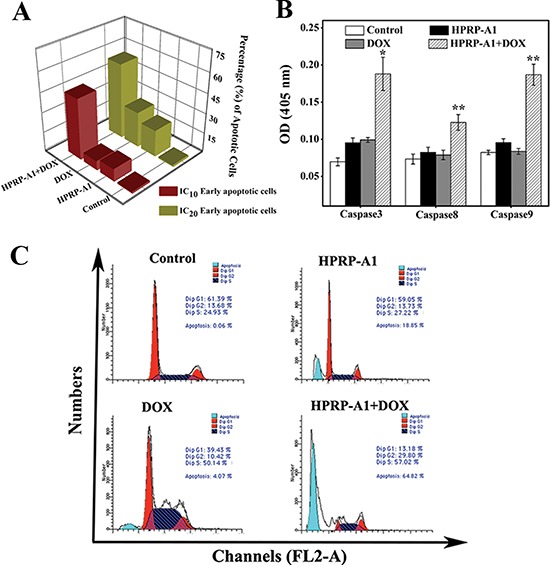
Apoptosis and cell cycle studies of HPRP-A1 and/or DOX *in vitro* **(A)** Percentage of early apoptotic cells, assessed by flow cytometry. HeLa cells were treated with HPRP-A1 and/or DOX at IC_10_ and IC_20_ concentrations (IC_10_ was 12 and 0.8 μg/ml, and IC_20_ was 16 and 1.2 μg/ml for HPRP-A1 and DOX, respectively). **(B)** Caspase-3, -8 and -9 activity. Cells were treated with HPRP-A1 (12 μg/ml) and/or DOX (0.8 μg/ml) for 24 h before measuring caspase activity levels. Data are the mean ± SD of three independent experiments. Statistical analysis compared the HPRP-A1+DOX treatment group with the HPRP-A1 and DOX alone treatment groups (*P < 0.05; **P < 0.01). **(C)** Cell-cycle phase distribution after treatment with HPRP-A1 (12 μg/ml, IC_10_) and/or DOX (0.8 μg/ml, IC_10_) for 24 h, analyzed by flow cytometry. Image shown is representative of three independent experiments.

To investigate the effect of combination therapy on cell apoptosis, HeLa cells were incubated with HPRP-A1, DOX or HPRP-A1+DOX for 24 h. Flow cytometric analysis of Annexin V/PI staining of cells treated with HPRP-A1+DOX at IC_10_ concentrations (HPRP-A1 12 μg/ml, DOX 0.8 μg/ml) revealed synergistically (rather than summatively) increased early apoptosis (45.70%) compared with each compound alone (HPRP-A1: 9.27%; DOX: 6.71%) (Fig. 4A). The synergistic trend was also apparent at IC_20_ concentrations (HPRP-A1 16 μg/ml, DOX 1.2 μg/ml), where the combination treatment resulted in 56.29% of cells being in the early apoptosis phase compared with only 19.86% and 26.40%, respectively, with HPRP-A1 and DOX treatment alone. The activities of caspase-3, -8 and -9 were tested using the corresponding caspase activity detection kits, and Fig. 4B shows that these enzymes were minimally activated by each agent alone but were strongly activated by the combination therapy.

### Cell cycle analysis

HPRP-A1 and DOX trigger G0/G1 and G2/M arrest in HeLa cells, respectively (data not shown). As showed in Fig. 4C, HeLa cells treated with the two drugs together for 24 h resulted in an increase in sub-G1 arrest (64.82% compared with 4.07% in DOX) and in G2/M arrest (29.80% compared with 10.42% in DOX). These findings also strengthened the increased caspase-3 activity and cell apoptosis population observed after the treatment with the combination therapy (Fig. 4A & 4B).

### HPRP-A1/DOX combination inhibits HeLa cell growth *in vivo*

To evaluate this synergistic antitumor action *in vivo*, mice were inoculated with HeLa cells (1 × 10^6^ in 100 μl PBS) subcutaneously into the right armpit. After tumors had grown to about 200 mm^3^ (~10 days), the animals were divided into four groups such that weight and tumor-size differences among the groups were minimized. In the next 15 days, the animals were given DOX (1 mg/kg weight, based on the minimum side effects [4]) and/or HPRP-A1 (10 mg/kg weight, based on the IC_50_ ratio of HPRP-A1/DOX) once every two days by direct injection into the tumors. PBS was given as the control. After this time (25 days total), mice were sacrificed and the tumors collected.

**Figure 5:**
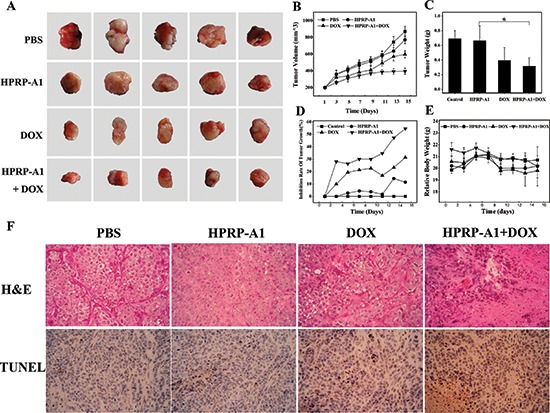
Effect of HPRP-A1 and DOX combination on inhibiting HeLa cell xenograft growth in nude mice **(A)** Tumor pictures. **(B)** Tumor growth curves by treatment group (*n* = 5 mice/group). **(C)** Average tumor weight at termination of study. **(D)** Percentage inhibition of tumor growth. **(E)** Body weight measured during the experimental period. Data are mean ± SD. **(F)** Double-stained HeLa cell xenograft specimens. Images of a section from each group were stained with H&E assay (upper panels) and TUNEL assay (lower panels).

Fig. 5A and 5B reveals marked differences in tumor volume at the end of the experiment. Tumor volumes in the combination group (395.40 mm^3^) were significantly smaller than those in the control (870.86 mm^3^), HPRP-A1 alone (771.29 mm^3^) and DOX alone (597.17 mm^3^) groups. Fig. 5C shows that the average tumor weight in the combination group was lower than in the control, HPRP-A1, and DOX groups (0.32 g *vs*. 0.69 g, 0.66 g, and 0.39 g, respectively). Fig. 5D clearly shows that mice treated with the combination therapy experience much greater tumor growth inhibition (54.60%) than animals treated with DOX alone (31.43%) or HPRP-A1 alone (11.43%). There was no obvious body weight change in any of these groups at the end of the *in vivo* study (Fig. 5E). Thus, combining low-doses of HPRP-A1 and DOX produces significant antitumor effects *in vivo*. Histological images using hematoxylin-and-eosin (H&E) staining showed that after applying HPRP-A1+DOX, a massive cancer cell remission occurred in the tumor tissue, whereas no such changes were apparent in the groups treated with DOX or HPRP-A1 alone (Fig. 5F). Moreover, images obtained using an *in situ* TUNEL assay showed that cell apoptosis was highest in the tumor tissue harvested from the mice treated with HPRP-A1+DOX, further confirming that the synergistic action of this combination therapy at inhibiting tumor growth is due to enhanced cellular apoptosis (Fig. 5F).

## DISCUSSION

HPRP-A1 and HPRP-A2 are amphipathic α-helical peptides with potent antimicrobial activity against various Gram-positive and Gram-negative bacteria and negligible hemolytic activity against human red blood cells [20]. Here, these peptides have been demonstrated to possess significant anticancer activity in two cancer cell lines. These α-helical membrane-active peptides disrupt the target cell membrane by a necrotic mechanism at high concentrations [22]. However, we have demonstrated here that, at lower concentrations, they induce target cell apoptosis. In addition, these peptides assist other chemical drugs in passing the cytoplasmic membrane, thus concentrating them within their intracellular targets.

In the *in vitro* and *in vivo* study, we have demonstrated that co-application of HPRP-A1 or HPRP-A2 with either DOX or EPI produces synergistically increased growth inhibition of HeLa and HepG2 cancer cells by enhancing apoptosis. All Q values were >1.15, which indicates the synergy of combined therapy *in vitro*. Our data further suggested that the HPRP-A1 enhancement of DOX-mediated apoptosis appeared to be caspase-dependent and involved both the extrinsic and intrinsic parts of the caspase cascade in HeLa cells. Through cell cycle analysis and flow cytometry, we also showed that cell apoptosis was correlated with the induction of cell cycle arrest in G2/M, as it is when using DOX alone. We also used a HeLa mouse xenograft model to produce *in vivo* confirmation that the HPRP-A1/DOX combination has synergistically higher antitumor activity than HPRP-A1 and DOX used alone, validating the clinical potential of this combined therapy.

It is interesting to see that, compared with DOX alone, the volume of tumor treated by HPRP/DOX decreased significantly, while the weight of tumor did not change as much as the tumor volume (Fig. 5B and 5C). This phenomenon may be attribute to the fact that cell apoptosis can readily decrease the volume of the cell but not the weight, since one of the characteristics of cell apoptosis is apoptotic shrinkage and detachment of cells [23] and cells are not totally degraded at this time. Hence, the changes of the tumor volume are greater than those of the tumor weight. The tumors from mice treated with HPRP-A1+DOX had more apoptotic cells than those from mice treated with control, HPRP-A1 alone or DOX alone (Fig. 5F). Moreover, H&E staining of tumor sections also demonstrated the superior efficacy of the combination therapy in inhibiting tumor proliferation (Fig. 5F). Our previous study showed that α-helical membrane-active peptides produce pores or channels in eukaryotic cell membranes and bind quickly to the surface of HeLa cells *via* a strong electrostatic interaction [22]. This previous study concluded that the sole target of HPRP-A1 was the cytoplasmic membrane because it exhibits a broad spectrum of antibacterial and antifungal activities [20]. Normal cell membranes, such as those in red blood cells, are characterized by zwitterionic phospholipids. By contrast, cancer cells have more anionic phospholipids in their outer leaflet, much like prokaryotic cell membranes [24–26]. In addition, many cancer cell membranes contain O-glycosylated mucin, a type of glycoprotein which increases the negative charge on the cancer cell surface [27]. The resultant increase in electrostatic interaction between cationic anticancer peptides and the negatively-charged cancer cell surface, together with the selectivity of these peptides for cancer cell membrane components, contributes to their high selectivity for cancer cells over healthy eukaryotic cells [12, 19]. In addition, there is a higher number of microvilli on cancer cells than normal cells [28], which increases the membrane surface area and thus the concentration of bound peptide on the cancer cell surface [24, 29]. This study thus confirms that the HPRP-A1/HPRP-A2 target is the cancer cell membrane, in stark contrast to the mechanism of DOX anticancer activity which is believed to involve DNA damage through topoisomerase II inhibition and free radical generation *via* a redox reaction [30, 31]. The therapeutic efficacy of many anticancer drugs is limited by their poor penetration into tumor tissue, which limits the doses of drugs that can be safely administered to cancer patients, leading to reduced efficacy and the development of drug resistance. A study on mouse tumor models shows that a previously characterized tumor-penetrating peptide, iRGD, increased vascular and tissue permeability in a tumor-specific manner, allowing coadministered drugs to penetrate into extravascular tumor tissue. Systemic injection with iRGD improved the therapeutic index of drugs of various compositions, including a small molecule doxorubicin [8]. We believe, in this study, the HPRP-A1 and HPRP-A2 increase the membrane permeability and allow DOX/EPI to penetrate into tumor cells like iRGD; moreover, the peptides can cause the death of tumor cells simultaneously, which is superior to iRGD.

The synergy shown here permits the use of relatively low concentrations of peptides and drugs (<IC_20_) to achieve significant anticancer effects *in vitro* and *in vivo*. This dose reduction minimizes drug effects on normal cells, enabling an effective apoptosis-mediated anticancer effect without inducing harmful adverse events. In addition, because HPRP-A1 and HPRP-A2 were equally effective at potentiating the effects of DOX/EPI, we are confident that the synergy does not depend on the stereo-isomeric structure.

## MATERIALS AND METHODS

### Peptides

α-Helical peptides HPRP-A1 and HPRP-A2 (peptide sequence Ac-FKKLKKLFSKLWNWK-amide, consisting of all L- or all D-amino acids, respectively) were synthesized by solid-phase methods using Fmoc (9-fluorenyl-methoxycarbonyl) chemistry as described previously [20] and purified (>95% purity) by RP-HPLC. Further characterization was by mass spectrometry and amino acid analysis. Doxorubicin hydrochloride (DOX·HCl) and epirubicin hydrochloride (EPI·HCl) were purchased from Meilun Biology Technology Co., Ltd. (Dalian, China).

### Cell culture

Human cervix carcinoma cells (HeLa) and human hepatocellular carcinoma cells (HepG2) cells obtained from the American Type Culture Collection which authenticates the cell lines by short-tandem repeat DNA testing, were used within 6 months of resuscitation and grown in DMEM with fetal bovine serum (FBS; 10% v/v), penicillin (100 U/ml), and streptomycin (100 U/ml) in a humid atmosphere at 37°C with 5% CO_2_.

### Cell viability assay

HepG2 and HeLa cells (8 × 10^3^) were plated in triplicate in a 96-well microtiter plate. Complete medium was replaced after 24 h with 100 μl of fresh medium containing various concentrations of drugs. After a further 24 h, cells were incubated with MTT at 37°C for 4 h. Thereafter DMSO was added to dissolve the formazan crystals and the absorbance at 492 nm was measured with a microplate reader (GF-M3000; Gaomi Caihong Analytical Instruments Co., Ltd. Shandong, China). Jin's formula was used to further quantify the synergistic effect of the combination treatment of HPRP-A1 and DOX. The formula is: Q = Ea+b / (Ea + Eb − Ea × Eb), where Q is the combination index; Ea + b represents the cell proliferative inhibition rate of the combined drug; Ea and Eb are signs of the cell proliferative inhibition rate of each drug. After calculation: *Q* < 0.85, *Q* > 1.15 and 0.85 < *Q* < 1.15 indicate antagonism, synergy, and additive effect, respectively.

### Hemolytic activity

Peptide or drug samples were serially diluted in PBS in round-bottomed 96-well plates to give a volume of 70 μl sample solution in each well. Human erythrocytes anticoagulated with EDTA were collected by centrifugation (1000 × g) for 5 min, washed twice with PBS, then diluted to a concentration of 2% in PBS. Erythrocytes (70 μl of 2% suspension) were added to each well to give a final concentration of 1% human erythrocytes in each well, and plates were incubated at 37°C for 1.5 h. The plates were then centrifuged for 10 min at 3000 rpm and the supernatant (90 μl) transferred to a flat-bottomed 96-well plate. The release of hemoglobin was determined by measuring the absorbance of the supernatant at 540 nm. Hemolytic activity was determined as the minimal peptide concentration to cause hemolysis. Erythrocytes in PBS and distilled water were used as negative (0%) and positive (100%) hemolysis controls, respectively.

### Flow cytometric analyses

HeLa cells (1 × 10^6^ cells/well) were seeded in six-well plates. DOX (1.0 μg/ml) or/and HPRP-A1 (12 μg/ml) were then added to each well. After incubation, the cells were collected at different time intervals (2, 4, 6 and 24 h) for measurement of doxorubicin fluorescence using flow cytometry (FACSCalibur, Becton-Dickinson, San Jose, CA, USA).

### Fluorescence microscopy

Cells were treated with DOX and/or HPRP-A1 for different time intervals (1 and 3 h) at a final DOX concentration of 15 μg/ml in basal DMEM. Untreated cells were used as a control. Cells in a 6-well plate were harvested, and washed three times with PBS before being fixed with 70% ice-cold ethanol for 5 min at 4°C. Cell nuclei were then stained with 4, 6-diamidino-2-phenylindile (DAPI, blue) for 30 min at room temperature. Images of cells were obtained by fluorescence microscopy (Olympus, Japan). All images are at 400× magnification.

### Scanning electron microscopy

HeLa cells were seeded in a 24-well plate with sterilized cover slips and incubated overnight. HPRP-A1 and/or DOX (IC_20_ concentrations) were added the following day and the plate was incubated at 37°C for 24 h. Preparation of cells for electron microscopy was performed as described previously [22] and cells were analyzed using an S3400 scanning electron microscope (Hitachi, Japan).

### Apoptosis assay

Apoptosis of HeLa cells was detected using the Annexin V-FITC Apoptosis Detection Kit (BD Biosciences). HeLa Cells (1 × 10^6^) were seeded in 6-well plates for 24 h. After induction with HPRP-A1 or/and DOX (IC_10_ and IC_20_ concentrations, respectively) for 24 h, the cells were collected. Subsequent procedures were performed according to the manufacturer's protocol. AnnexinV-FITC apoptosis detection was by flow cytometry. Activity of caspase-3, -8 and -9 was tested using the corresponding caspase activity detection kits (BD Biosciences), according to the manufacturers' instructions.

### Cell cycle analysis

Cell cycle arrest was studied using flow cytometry. HeLa Cells (1 × 10^6^) were cultivated in 6-well plates containing 2 ml media and allowed to attach overnight at 37°C. Medium (2 ml) containing HPRP-A1 (12 μg/ml) and/or DOX (0.8 μg/ml) was then added to each well. Medium alone was used as a negative control. After incubation for 24 h, cell cycle distribution was determined by a FACScan cytometer and Cell Quest software (FACSCalibur, Becton-Dickinson). All experiments were performed in triplicate.

### Mouse tumor xenograft model

Six-week-old female BALB/c nude mice (obtained from Shanghai SLAC Laboratory Animal Co., Ltd. China) were housed in appropriate animal care facilities during the experimental period, and were handled in accordance with the Guide for Care and Use of Laboratory Animals in China. All animals were inoculated subcutaneously into the right armpit with 1 × 10^6^ HeLa cells in 100 μl PBS. After tumors had grown to about 200 mm^3^ (~10 d), the animals were divided into four groups such that weight and tumor-size differences between groups were minimized. Over the next 15 d, HPRP-A1 (10 mg/kg body weight) and/or DOX (1 mg/kg body weight) were given once every two days by direct injection into the tumors. Mice treated with PBS were used as controls (Supplementary Fig. S2). Tumor volumes [(major axis) × (minor axis)^2^ × 1/2] were measured at defined time periods. The inhibition rate of tumor growth (%) was calculated using the following formula: (tumor volume of control - tumor volume of experiment)/tumor volume of control × 100%. Mice were sacrificed and the tumors were collected for further immunohistochemistry and TUNEL assays.

### Statistical analysis

Average data are presented as the mean±SD of at least three independent experiments. Statistical significance of differences between groups were analyzed by t-test, with significance accepted at *P* < 0.05 (*) and *P* < 0.01 (**).

## SUPPLEMENTARY FIGURES


