# Supplementary material for: Two hits are better than one: synergistic anticancer activity of α-helical peptides and doxorubicin/epirubicin
Source: Oncotarget. 2014 Dec 19;6(3):1769–78. doi: 10.18632/oncotarget.2754 (PMC4359330; doi:10.18632/oncotarget.2754)
Supplement: Supplementary file 1 [file oncotarget-06-1769-s001.pdf]

## SUPPLEMENTARY FIGURES

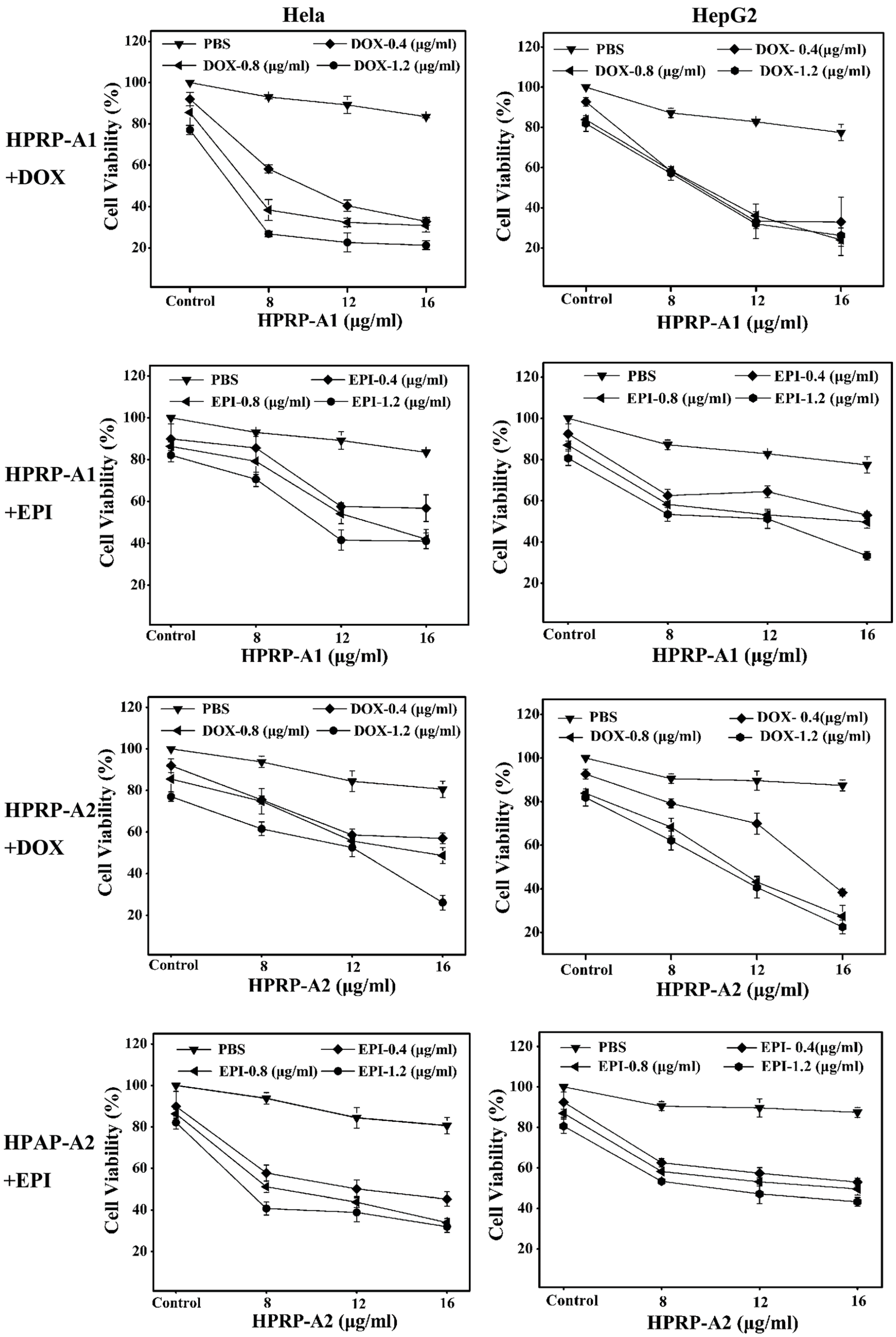


**Supplementary Figure S1: Growth inhibition in HeLa and HepG2 cells after incubation for 24 h with a combination of HPRP-A1/HPRP-A2 (16, 12, and 8 μg/ml) and DOX/EPI (1.2, 0.8 and 0.4 μg/ml).** Results are expressed as percentage of the control ± SD of three independent experiments.

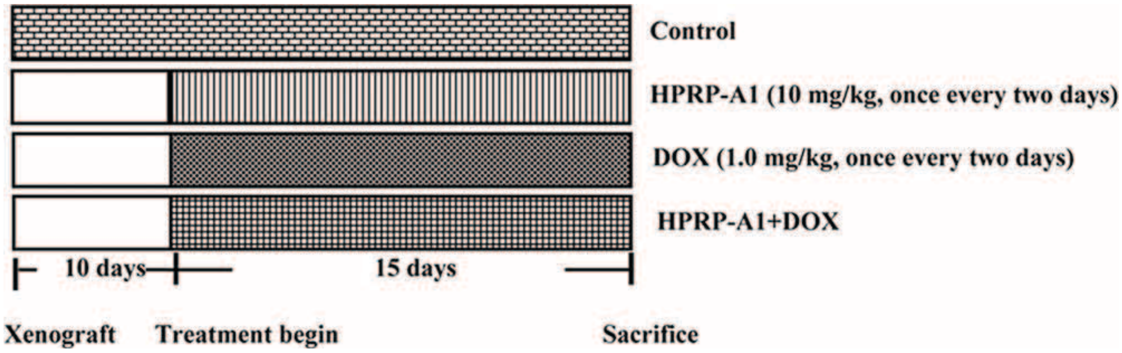


**Supplementary Figure S2: Experimental scheme of mouse HeLa xenograft model.** The mice were divided into four groups. Nude mice at the age of 6 weeks were injected with 1 x $10^6$ HeLa cells. After 10 days, HPRP-A1 (10 mg/kg body weight) and/or DOX (1 mg/kg body weight) were given once every two days by direct injection into the tumors. Mice treated with PBS were used as controls.
